# ISCEV extended protocol for the photoreceptor directed ERG using full-field silent substitution stimuli

**DOI:** 10.1007/s10633-026-10087-w

**Published:** 2026-02-22

**Authors:** Jan Kremers, Mirella T.S. Barboni, Andrew J. Zele, Beatrix Feigl, J.Jason McAnany, Anthony G. Robson, Balázs Vince Nagy, Neil Parry, Omar A. Mahroo, Cord Huchzermeyer

**Affiliations:** 1 Section for Retinal Physiology, Department of Ophthalmology, University Hospital Erlangen, Schwabachanlage 6, 91054 Erlangen, Germany; 2 Department of Ophthalmology, Semmelweis University, Budapest, Hungary; 3 Centre for Vision and Eye Research, Queensland University of Technology, Brisbane, Australia; 4 School of Medicine, Queensland University of Technology, Brisbane, Australia; 5 Queensland Eye Institute, Brisbane, Australia; 6 Department of Ophthalmology and Visual Sciences, University of Illinois Chicago, Chicago, IL USA; 7 Moorfields Eye Hospital, London, UK; 8 Institute of Ophthalmology, University College London, London, UK; 9 Department of Mechatronics, Optics and Mechanical Engineering Informatics, Faculty of Mechanical Engineering, Budapest University of Technology and Economics, Budapest, Hungary; 10 Vision Science Centre, Manchester Royal Eye Hospital, Manchester, UK; 11 School of Health Sciences, University of Manchester, Manchester, UK

**Keywords:** Electroretinography (ERG), Silent substitution, Photoreceptors

## Abstract

The International Society for Clinical Electrophysiology of Vision (ISCEV) Standard for full-field electroretinography (ERG) describes a minimum procedure, but encourages more extensive testing. This ISCEV extended protocol describes an extension to the full-field ERG Standard, namely the ERG responses to silent substitution stimuli that are directed at the response modulation of single photoreceptor types. The method can be used to detect selective defects in distinct photoreceptor types and post-receptoral retinal pathways. This document, based on existing literature, provides protocols for recording and analyzing the responses to a single class of photoreceptor directed stimuli. For each photoreceptor type (rods, L-, M- and S-cones), optimal luminance conditions and temporal frequencies for Ganzfeld recordings are provided. The exact stimulus conditions depend on the spectral properties of the stimulator. An accompanying technical report describes an app that is meant to provide support in calculating stimuli for individual stimulators.

**Supplementary Information:**

The online version contains supplementary material available at 10.1007/s10633-026-10087-w.

## Introduction

The International Society for Clinical Electrophysiology of Vision (ISCEV) publishes and updates open access standards for clinical tests of the visual system (see the ISCEV website www.iscev.org/standards). The Standard for full-field electroretinography (ERG) describes an established minimum protocol [1] used to evoke and record mass responses of the retina to flashes of light. In addition to the ISCEV Standard ERG, extended protocols may be useful to enhance patient management, or for detailed characterization. ISCEV extended ERG protocols currently include those for the photopic negative response [2], the dark-adapted red flash ERG [3], the photopic On–Off ERG [4], stimulus–response series for light-adapted full-field ERG [5] and for the dark-adapted full-field ERG b-wave [6], the S-cone ERG [7], and for the derivation and analysis of the strong flash rod-isolated ERG a-wave [8].

This extended protocol describes the photoreceptor directed ERG (pdERG) elicited using silent substitution stimuli. Photoreceptor directed stimulation enables study of the responses elicited by single photoreceptor types, including rods, S-, M- and L-cones and the melanopsin expressing intrinsically photosensitive retinal ganglion cells (ipRGCs), or by combinations of photoreceptor classes. The technique is gaining recognition as a possible adjunct to routine testing, using methods that are broadly accepted by experts in the field. The protocol was prepared by the authors in accordance with ISCEV procedures (https://iscev.wildapricot.org/standards) and was approved by the ISCEV Board of Directors on January 22, 2026. This document does not contain safety standards, standards for clinical care and management, or advice for patient privacy or data protection; users of this Guideline must choose instruments approved for clinical use in their jurisdiction and follow local and national requirements for clinical safety, care, healthcare information technology and data protection.

## Scope and applications

A pdERG allows isolated measurements of the global responses driven by any one of the five types of light sensitive cells within the retina, including three cone types, rods and melanopsin expressing ipRGCs. In addition, pdERGs can be used to study major post-receptoral (cone opponent and luminance sensitive) pathways. They may be used to complement ISCEV standard ERGs [1] in the diagnosis and characterisation of retinal dysfunction. Sensitivity and diagnostic value have yet to be fully established, but applications include the investigation of generalized cone and/or rod system or ipRGC dysfunction, with particular value in conditions that may entail selective or preferential involvement of one or more photoreceptor types or post-receptoral pathways. Typical applications and exclusions are outlined below.

- (a) Congenital color vision deficiency (CCVD). Genetically determined color vision losses can affect a single class of cone e.g., L-cones and M-cones are selectively affected in protanopia (OMIM: #303900) or deuteranopia (OMIM: #303800) respectively. Subjects with classic CCVD show normal ISCEV standard full-field ERGs, but pdERGs can be used to detect the absence of cone specific responses, thereby providing objective information regarding the color deficiency type. In addition, the absence of a functional L-M cone opponent pathway also leads to changes in the pdERGs in comparison to color-normal subjects. Importantly, however, in anomalous trichromacy the interpretation of pdERGs are confounded because the cone fundamentals in such cases strongly deviate from those used for calculating stimulus conditions (see below).
- (b) Inherited and acquired retinopathies. pdERGs can be used to investigate photoreceptor dysfunction in cases of inherited retinal dystrophy (IRD) and in acquired retinal disorders. For example, for probing rod function in retinitis pigmentosa (RP; rod-cone dystrophy), where a potential advantage is that rod-directed ERGs can be recorded at relatively high retinal illuminance, without the need for conventional dark adaptation, and in disorders primarily or preferentially involving cones. pdERGs may also be used to assess cone or rod photoreceptor dysfunction secondary to a locus that is post-phototransduction or inner retinal, for instance resulting from retinal re-modelling.

- (c) Application for neurological and systemic diseases. pdERG recordings can be used to evaluate the function of cone, rod and melanopsin expressing ipRGCs in optic neuropathies. For example, in glaucoma, ipRGCs, the L-cone and luminance pathways may be affected [9–11]. IpRGC function has been tested psychophysically and with pupillometry in neurological, psychiatric [12] and metabolic disorders [13].

## Patient population

Patients of all ages, able to tolerate Ganzfeld stimulation, referred for investigation of possible retinal dysfunction, especially those with suspected pathology preferentially or selectively affecting L-, M- or S-cones, rods or melanopsin expressing ipRGCs. Testing of anomalous trichromats should be avoided unless non-standard cone fundamentals that match the subject’s spectral sensitivities are used in the calculations.

## Technical issues

### Principles of silent substitution stimulation

The silent substitution procedure is based upon the principle of univariance according to which the excitation of a photoreceptor after an isomerization (i.e., the absorption of a photon) is uniform and independent of the wavelength of the absorbed photon. As a result, the wavelength and luminance of the stimulus can be interchanged without altering the photoreceptor excitations (i.e., the silent substitution). A measured response can then be attributed to the photoreceptor(s) whose excitation(s) change during the stimulus interchange. Photoreceptor-directed stimulation employs controlled modulation of the outputs of differently colored light sources (primaries) resulting in selective activation of one or more of the five photoreceptor types in the human eye (rods, S-, M- and L-cones and the melanopsin expressing ipRGCs) through silent substitution of the non-targeted photoreceptors’ output.

The selection of 3, 4 or 5 primaries enables silencing of two, three or four photoreceptor classes respectively [14]. Three primaries may be used to create cone isolating stimuli (rods and melanopsin expressing ipRGCs are not silenced); four primaries are used to obtain cone or rod isolating stimuli (melanopsin expressing ipRGCs are not silenced); five primaries are required to obtain complete functional isolation of rod, L-, M-, S-cone and melanopsin expressing ipRGCs. For routine clinical implementation, commercially available Ganzfeld stimulus sources that contain four primaries (nominally “blue,” “green,” “amber,” and “red” see supplementary material Fig. S1) can modulate the rods, S-cones, M-cones, and L-cones are acceptable. The melanopsin containing ipRGCs are not typically silenced. However, their contribution to the full-field ERG is expected to be minimal compared to the other photoreceptor types because they do not respond strongly to frequencies beyond about 2 Hz [15, 16].

The method of silent substitution is conducted with references to the five photoreceptor spectral sensitivities measured at the cornea (called fundamentals *F*_*P*_*(λ),* defined as the 10° CIE Standard Observer [CIE S-026 2018]; see supplementary material Fig. S2). The spectral power distributions of the available primary lights (*E*_*i*_*(λ)*) in the stimulator will determine the maximum achievable photoreceptor directed stimulus contrast [14].

### Calculation of the parameters

To obtain the excitations (*S*_*P*_) of each photoreceptor class *P*, their fundamentals *F*_*P*_*(λ)* are multiplied by the spectral power distribution of each primary (*E*_*i*_*(λ)*):

$${S}_{P}=\int {F}_{P}\left(\lambda \right)\bullet \left({E}_{1}\left(\lambda \right)+{E}_{2}\left(\lambda \right)+\dots +{E}_{N}\left(\lambda \right)\right) \bullet d\lambda $$

This is performed for each photoreceptor type. For calculations, the integral is approximated by the addition of the multiplication at discrete steps (i.e., numerical integration).

If two stimuli are exchanged the photoreceptor sensitivities (*S*_*P*_) need to be calculated before and after the exchange. If the two sensitivities are equal then the substitution is silent. This can be done for any stimulus waveform. For periodic stimuli in which the mean excitation equals the mean of the minimal and maximal excitation (such as sine, rectangular and sawtooth waveforms) the excitation change can be calculated by the Michelson contrast (*C*_*P*_):

$${C}_{P}=\frac{\left({S}_{P1}-{S}_{P2}\right)}{\left({S}_{P1}+{S}_{P2}\right)}*100\%$$

in which the *S*_*P*1_ and *S*_*P*2_ are the photoreceptor excitations elicited by the stimuli before and after the exchange. For aperiodic stimuli, such as flashes, Weber contrast (i.e., excitation due to the flash divided by excitation by the background) is the relevant metric.

The calculation of the excitation contrast (*α*) from contrast in primary output (*β*) is a matrix (*A*):$\alpha =\beta A$. The primary contrasts required to modulate a specific photoreceptor class (or a combination) can then be calculated by using the inverse of the A-matrix: $\beta =\alpha {A}^{-1}$. When generating photoreceptor directed stimuli under conditions of silent substitution, the contrasts of one or more photoreceptors are zero. If the contrasts of all but one photoreceptor type are zero, then the stimulus modulates the response of the photoreceptor type with non-zero contrast. These stimuli are the pd-stimuli for ERG recordings. When the number of primaries in a system is less than the number of photoreceptor classes, the untargeted photoreceptor is removed from the corresponding A-matrix and the change in primary outputs will generally introduce a concomitant photoreceptor contrast in the untargeted photoreceptor as a confounding factor [14].

Measurements of the absolute spectral power distributions of the primaries are essential to calculate the sensitivities of the different photoreceptor classes and the conditions. This requires a spectroradiometer that gives the output in W.m^−2^.sr^−1^.nm^−1^) as a function of wavelength (spectral radiance). The total radiance of a stimulus is obtained by the integration over wavelength. As most commercial systems specify stimuli in luminance (cd.m-2), a conversion between radiance and luminance is considered in our calculations. Light sources where spectral distributions are not constant (for instance at different mean luminance levels or due to electric power or operating temperature changes) should be avoided.

### Stimulus waveform, temporal frequency and mean luminance

Silent substitution studies have employed stimuli modulated according to different temporal waveforms such as a sawtooth or a sine wave. The advantage of sinusoidal stimuli is that the responses can be Fourier analyzed (see Section “Response evaluation”) and the amplitudes and timing at the stimulus frequency and harmonics can be precisely extracted, thereby ignoring disturbances e.g., caused by mains frequency interference. In addition, the responses at different temporal frequencies can be correlated with activities in different post-receptoral pathways. At intermediate temporal frequencies (8–16 Hz), L- and M-cone driven pdERGs reflect properties of the red-green chromatic (L-M cone opponent) channel, thereby enabling studies on color vision in basic research and clinical settings. At high temporal frequencies (> 30 Hz), L- and M-cone driven pdERGs reflect activity of the luminance pathway, so that function and dysfunction of luminance vision can be independently investigated [17, 18]. The S- cone driven pathway has relatively low temporal resolution and has been studied with pdERGs using a relatively low temporal frequency (at about 6 Hz) [19, 20]. Rod driven ERGs can be reliably measured up to about 8 Hz [21, 22]. To avoid interference with low frequency disturbances (such as those caused by eye movements) it is recommended to use temporal frequencies above 4 Hz.

For cone-driven responses, a photopic mean luminance is recommended. A mean luminance at about 300 cd.m^−2^ has been found to achieve good results [20]. A lower luminance is necessary for obtaining rod-driven responses. Experiments have shown that a luminance of about 0.2–2 cd.m^−2^ can give good rod-driven responses without the need for long dark adaptation periods [21]. It should be taken into account that the state of adaptation in the non-modulated photoreceptors may influence the measured response [23, 24]. Therefore, the chromaticity of the stimulus should also be given.

### Limitations based on a standard observer

The pdERG method is limited by differences between individuals relative to the Standard Observer. This can result from opsin gene polymorphisms and differences in transmission in ocular media. As a consequence, the observer’s *A*-matrix deviates from that of the Standard Observer (note that similar limitations apply to the methods in which photoreceptor responses are recorded with desensitizing backgrounds). The sensitivities of the photoreceptors depend on retinal location. Additionally, local factors such as the retinal vasculature may block incident radiation, and macular “luteal” pigment may absorb shorter wavelength light, potentially influencing responses to focal or spatially restricted stimuli. The use of full-field stimuli largely negates the effects of focal factors, irrespective of the spectral content of the light.

Within limits (excluding anomalous trichromats), observer calibrations have been performed to rescale the *A*-matrix [14, 15] to partly reduce the effects of inter-individual differences, but such methods are beyond the scope of this protocol.

## Protocol specifications

The procedures for patient preparation and recording parameters (including filter settings) are as specified by the ISCEV Standard for the clinical full-field ERG [1] including the use of Ganzfeld stimulation after mydriasis. Additional specifications are listed below:

- (a) Selection of primaries
  Three, four or five primaries may be used, intended for double, triple or quadruple silent substitution respectively, although the number possible may be limited by the Ganzfeld stimulus source. If 3 or 4 primaries are used, the contrast of the modulated photoreceptor class or classes must be reported. The characteristics of the primaries may be selected according to the online application (SilentSubstiTutor; see below), although the use of this resource is optional for compliance with this protocol.

- (b) Stimulus strength and temporal characteristics.
  Stimuli are modulated according to a sinusoidal waveform. Luminance and temporal frequency must be adjusted depending on the intended target photoreceptor type or target visual subsystem under investigation (e.g., Table 1). The excitation contrasts in the different photoreceptor classes should be specified as Michelson contrast. If fewer than five primaries are employed, then the contrasts in the non-targeted photoreceptors should also be specified. Note that the mean luminance and temporal frequencies provided in Table 1 are based on published studies that have reliably elicited pdERGs, but the technique is highly flexible and applicable across a range of mean luminances and temporal frequencies.

**Table 1:** Examples of stimulus parameters suitable for full field cone and rod directed sinusoidal stimuli. Melanopsin expressing ipRGC-directed ERGs have only been recorded with temporal white noise stimuli [25] and are not included in the table

Photoreceptor type (and post-receptoral pathway)	Number of primaries	Mean luminance (cd.m^−2^)	Photoreceptor contrast*	Temporal frequency (Hz)	References
L- and M-cone; luminance pathway	3 or more	300	Identical for L- and M-cones (typically about 20%)	30–40	[20, 26]
L- and M-cone; red-green chromatic pathway	3 or more	300	Identical for L- and M-cones (Typically about 20%)	< 14	[20, 26]
S-cone	3 or more	300	Maximum possible contrast (can reach 70% or more)	6	[20]
Rod	4 or more	2	Maximum possible contrast (can reach 30%)	8	[21, 22]

*photoreceptor contrasts are based upon achievable contrasts with current commercially available stimulators

- (c) Light adaptation
  Sufficient time for adaptation to the specific background should be included e.g., in a light-adapted subject one minute of exposure to the mean luminance (of 300 cd.m^−2^) and mean chromaticity is sufficient. If performed after dark adaptation (for instance after a DA standard ERG), then 10 min of light adaptation to the mean luminance and chromaticity is likely necessary.

## Specification of primaries using the ISCEV online application

An online application (SilentSubstiTutor) for calculating the characteristics of the primaries necessary for obtaining a desired photoreceptor contrast can be found at the following website: https://huchzi.shinyapps.io/silentSubstitutor (app created by author CH). The specified link returns Michelson contrasts of the primaries for obtaining silent substitution conditions for sinusoidal (and other periodic) stimuli."Calculation of silent substitution stimuli for full-field electroretinography using the SilentSubstiTutor application" by Huchzermeyer and Kremers. A full description of the app can be found in an accompanying paper.

As a first step, the emission spectra of the primary lights of the stimulator must be selected. Alternatively, spectra of 3–5 primaries can be imported via a CSV file under “Advanced Settings.” A detailed description of how the CSV should be formatted is found in the app. Next, the mean luminance (averaged over time) of the primaries can be chosen. The possible luminance ranges can also be adjusted in the “Advanced Settings,” which may be saved by bookmarking the corresponding browser link.

The application then displays the resulting mean chromaticity (as CIE 10° x, y coordinates) and an estimated corresponding color in an unreferenced RGB space (for visualization only). On the subsequent screen, the user specifies the desired photoreceptor contrasts. The number of controllable photoreceptors depends on the number of primaries: with 3 primaries, modulation in the L-, M-, and S-cones can be controlled; with 4 primaries, rod signals can also be targeted; and with 5 primaries, melanopsin responses can additionally be controlled. Note that the excitation of photoreceptors that are not silenced may contribute to the ERG (which should be reported). Other settings, such as a suppressing adaptation (for rods) or temporal frequency (for rods and melanopsin expressing ipRGCs), may be used to minimise response intrusions.

The table summarizes key stimulus properties, including Michelson contrast, plus a plot showing primary light luminance as a function of time, assuming sinusoidal modulation. Desired contrasts may exceed 100% and are highlighted in red to warn the user that the condition cannot be achieved. The “Maximize” function scales all contrasts to their maximal achievable values such that the strongest contrast reaches 100%. The MaxLuminance entry in the table indicates the maximum luminance each primary will reach; values exceeding predefined luminance limits are also marked in red.

The final screen enables exploration of how deviations from standard assumptions affect photoreceptor isolation. The user can modify parameters such as the peak absorption wavelengths of L- and M-cones, lens age (in years) and macular pigment optical density (MPOD). However, it is emphasized that to comply with this protocol, MPOD should be specified as zero, as full-field responses reflect generalized retinal function, with a negligible contribution from the macula. In cases of double or triple silent substitution, the resulting contrasts in uncontrolled photoreceptors are also reported.

A short user manual is provided in the app. Version updates will be documented. When using the app, both this extended protocol and the app (with version number) should be referenced in the publication.

## Response evaluation

**Fig. 1:**
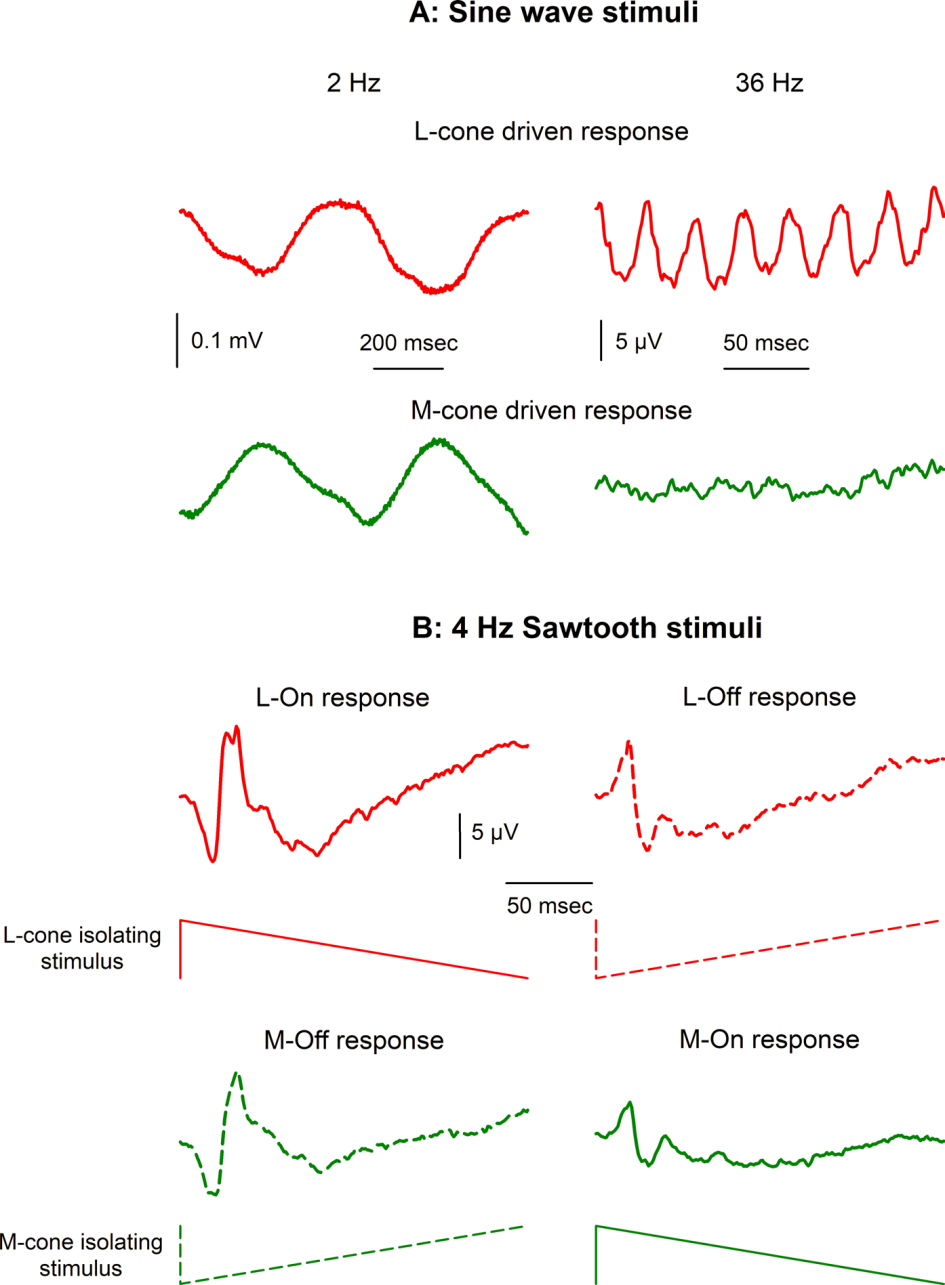
Responses to L- and M-cone directed full field stimuli using four primaries as specified in supplementary material Fig. S1. The mean chromaticity (in CIE 1931 color space) was x = 0.5951, y = 0.3857. The L- or M-cone directed contrasts were 18%. The contrasts in the non-targeted cones and the rods were 0% per definition; contrasts in the melanopsin expressing ipRGCs were −5.1% and 1.2% in M- and L-cone directed stimulation respectively (the negative sign indicating that the melanopsin expressing ipRGCs modulated in counter-phase with the cone modulation).** A** responses to 2 Hz (left) and 36 Hz (right) temporal frequency. Upper (red) plots: L-cone directed ERGs; lower (green) plots: M-cone directed ERGs. Adapted from [20].** B** Responses to 4 Hz sawtooth stimuli measured in a different subject. Upper left: rapid L-On sawtooth; upper right: rapid L-Off; lower left: rapid M-Off; lower right: rapid M-On. Sketches of the stimuli are given below the responses. Observe that L-On and M-Off responses resemble each other. Similarly, L-Off and M-On responses resemble each other, indicative of cone opponent post-receptoral signal processing. Averaged over 160 sweeps of 1 s each; filter settings between 0.5 and 300 Hz. Adapted from [27]

Figure 1 displays typical L- and M-cone directed ERGs with sinusoidal and sawtooth wave forms (sawtooth stimuli are not part of the extended protocol but are included to illustrate that cone opponency can influence ERG recordings).

**Fig. 2:**
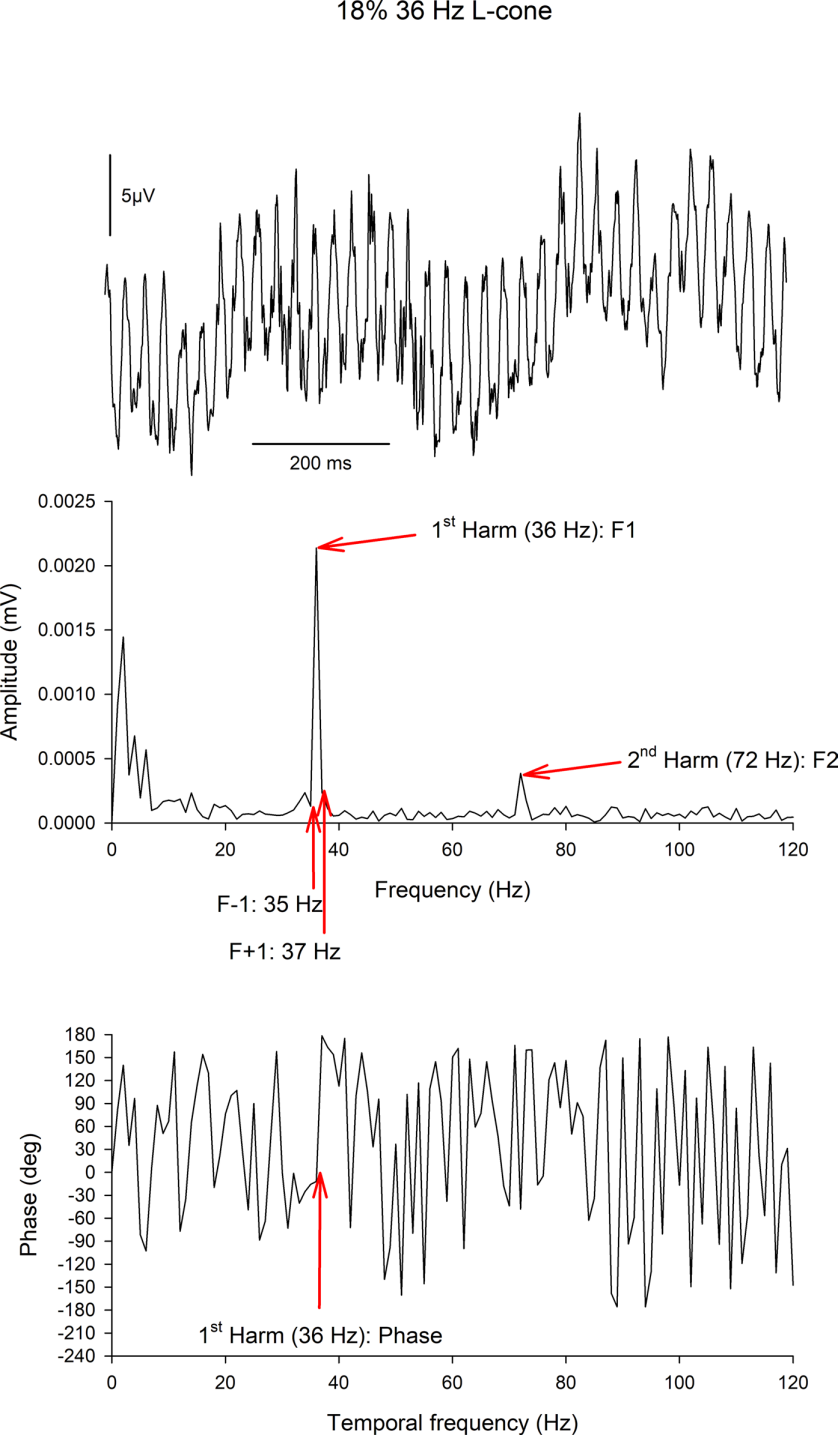
Visualization of the analysis with Fast Fourier Transform (FFT) of L-cone directed ERGs (18% cone contrast, 36 Hz) obtained from a typical subject during the same measurement used for the responses shown in Fig. 1A (upper right red traces). The upper plot shows the original response in a 1 s period (averaged over 40 sweeps). Low frequency changes can be observed to overlie the 36 Hz response. The FFT returns the amplitude (middle plot) and the phase (lower plot) as a function of temporal frequency. The amplitude plot shows a clear peak at 36 Hz (1^st^ harmonic or fundamental component; F1) and a smaller peak at 72 Hz (2^nd^ harmonic component; F2). The low frequency changes (below about 10 Hz) are clearly separate and do not interfere with the 36 Hz peak. At frequencies not related to the stimulus, the amplitudes are determined by noise. Noise for the 1^st^ harmonic component is defined as the average of the amplitudes at F1 + 1 (in this case 37 Hz) and F1-1 (35 Hz) [28]. The SNR is the amplitude at F1 divided by the noise (in this case SNR is about 11.5). If the SNR is larger than about two, the phase at F1 (red arrow in lower plot) is meaningful. The phases at frequencies that are represented by noise only are meaningless. Observe that the FFT only gives phases between −180° and + 180°. Actual phases can differ by integer multiples of 360° from the displayed phase

Evaluation of the responses depends on stimulus type. For sinusoidal stimuli, the responses are typically Fourier analyzed and amplitudes and phases of first (fundamental; at the stimulus frequency) and second (twice stimulus frequency) harmonics are extracted. Alternatively, the peak-to-trough amplitude and peak time can be calculated from the waveform, consistent with the ISCEV Standard for full-field ERG. An advantage of Fourier analysis is that an estimate of noise can be obtained by averaging the amplitudes at neighboring frequencies [28]. Harmonic phase should be discarded if the signal to noise ratio (SNR; i.e., the harmonic amplitude divided by noise amplitude) is smaller than a predetermined criterion. Generally, a SNR above three is sufficient. With SNRs between two and three, phase data can be variable but still valid. Figure 2 visualizes the extraction of the important parameters.

## Reporting

The pdERG extended protocol has the versatility to allow stimulation of different photoreceptor classes, requiring the use of widely different stimulus parameters, and comprehensive reporting of methods is essential to facilitate comparison between reports. Reporting of pdERGs follows the recommendations of the ISCEV full-field Standard ERG [1], and should also include the information below.

1. Type of equipment and light source used, and controlling software. If the SilentSubstiTutor application (above) was used, then this should be acknowledged.
2. Mean luminance (cd·m^−2^) of the full-field stimuli.
3. Chromaticity (CIE coordinates) of stimuli.
4. Emission spectrum of each primary (including the peak emission wavelength, λ_max_, and the full width at half maximum, FWHM, if the emission spectrum is symmetric); contrast (of each of the primaries and in each photoreceptor type).
5. Stimulus waveform and temporal frequency. The use of sine wave modulation should be stated (to comply with this protocol), as should the use of any additional temporal profiles.
6. Pupil diameters after mydriasis
7. Time of adaptation e.g., in darkness or to a specified background light.
8. The type and position of recording electrode used e.g., if silver thread, lower eyelid or lower fornix.
9. Known color vision deficits of the participant/s being tested should be documented.
10. As a minimum, data should be described based on Fourier analysis and measurement of the first (fundamental) and second harmonic amplitude, and SNR. The phase of the harmonics should be reported when the SNR is equal or larger than the predetermined criterion.

## Supplementary Information

Below is the link to the electronic supplementary material.

Supplementary file1 (DOCX 56 kb)

## Data Availability

I don't see a data availability statement. I don't know why this query was inserted at this place. No data arepresented that can be made available.
