# Correction: ISCEV extended protocol for the photoreceptor directed ERG using full-field silent substitution stimuli

**DOI:** 10.1007/s10633-026-10101-1

**Published:** 2026-03-26

**Authors:** Jan Kremers, Mirella T.S. Barboni, Andrew J. Zele, Beatrix Feigl, J.Jason McAnany, Anthony G. Robson, Balázs Vince Nagy, Neil Parry, Omar A. Mahroo, Cord Huchzermeyer

**Affiliations:** 1 Section for Retinal Physiology, Department of Ophthalmology, University Hospital Erlangen, Schwabachanlage 6, 91054 Erlangen, Germany; 2 Department of Ophthalmology, Semmelweis University, Budapest, Hungary; 3 Centre for Vision and Eye Research, Queensland University of Technology, Brisbane, Australia; 4 School of Medicine, Queensland University of Technology, Brisbane, Australia; 5 Queensland Eye Institute, Brisbane, Australia; 6 Department of Ophthalmology and Visual Sciences, University of Illinois Chicago, Chicago, IL USA; 7 Moorfields Eye Hospital, London, UK; 8 Institute of Ophthalmology, University College London, London, UK; 9 Department of Mechatronics, Optics and Mechanical Engineering Informatics, Faculty of Mechanical Engineering, Budapest University of Technology and Economics, Budapest, Hungary; 10 Vision Science Centre, Manchester Royal Eye Hospital, Manchester, UK; 11 School of Health Sciences, University of Manchester, Manchester, UK

**Correction to: Doc Ophthalmol** 10.1007/s10633-026-10087-w

In this article, the title was incorrectly given as ‘ISCEV extended protocol for the photoreceptor directed ERG using full-field silent substitution stimuli identification’ but should have been ‘ISCEV extended protocol for the photoreceptor directed ERG using full-field silent substitution stimuli’.

The original article has been corrected.

