# Supplementary material for: ISCEV extended protocol for the photoreceptor directed ERG using full-field silent substitution stimuli
Source: Doc Ophthalmol. 2026 Feb 22;152(2):247–56. doi: 10.1007/s10633-026-10087-w (PMC13083440; doi:10.1007/s10633-026-10087-w)
Supplement: Supplementary file 1 — Supplementary file1 (DOCX 56 kb) [file 10633_2026_10087_MOESM1_ESM.docx]

Supplementary material Fig. 1: Spectral output in [W/(sr.m².nm)] of a four primary stimulator used for the measurements, results of which are shown in Fig. 1. The curves are normalized to have the same areas under the curve of one W/(sr.m²). Peak wavelengths (FWHM) are: blue: 470 (22) nm; green: 524 (36) nm; orange: 594 (15) nm; red: 638 (19) nm. The mean luminances of the primaries are given.

Supplementary material Fig. 2: Fundamentals of the four photoreceptors and the melanopsin containing ipRGCs, normalized to unity at the maximal sensitivity.
